# Case report: Savolitinib induced severe adverse reactions resembling septic shock in an HIV-1–positive patient with advanced non-small cell lung cancer

**DOI:** 10.3389/fphar.2023.1089184

**Published:** 2023-02-02

**Authors:** Ye Xiong, Qing Cao, Yongzheng Guo, Xiang Liu, Xueling Zhu, Bohao Dai, Biao Zhu

**Affiliations:** Department of Infectious Diseases, State Key Laboratory for Diagnosis and Treatment of Infectious Diseases, National Clinical Research Center for Infectious Diseases, Collaborative Innovation Center for Diagnosis and Treatment of Infectious Diseases, The First Affiliated Hospital, School of Medicine, Zhejiang University, Hangzhou, China

**Keywords:** shock, adverse drug rection, targeted therapy, savolitinib, HIV-1

## Abstract

Savolitinib, a small-molecule inhibitor of the receptor tyrosine kinase mesenchymal-epithelial transition (MET) factor, was approved for the treatment of non-small cell lung cancer (NSCLC) by the China National Medical Products Administration in June 2021. Its safety for NSCLC treatment has been confirmed in several prospective cohort studies. Herein, we report a rare case of shock, a serious adverse event, after treatment with savolitinib in an HIV-1–positive patient with advanced NSCLC. A 38-year-old man with an 8-year history of HIV-1 positivity was diagnosed with NSCLC 5 years ago; the lung cancer recurred after surgical resection. Despite chemotherapy, immunotherapy, and targeted therapy, tumor progression continued. He received savolitinib because of MET amplification. In the first 2 weeks of savolitinib use, he developed a mild rash on his trunk. In the following month, he was hospitalized for fever and circulatory shock thrice after taking savolitinib 400 mg. He had no urticaria or eosinophilia. During the three hospitalizations, he was negative for pathogens. His condition gradually improved after treatment with antibiotics, steroids, and vasopressors. Attention should be paid to the occurrence of septic shock-like presentations when using savolitinib in HIV-1 patients with NSCLC.

## Introduction

Mesenchymal-epithelial transition (MET) or hepatocyte growth factor receptor (HGFR) is a member of the receptor tyrosine kinase family, which combines with its ligand HGF to regulate basic cell functions (Tovar and Graveel, 2017; Wood, 2021). However, abnormal MET gene amplification can lead to a wide range of human cancers (Grundy and Narendran, 2022). Thus, the HGF/MET axis is a feasible target for the development of cancer drugs. In recent years, MET inhibitors have been evaluated in preclinical investigations and clinical trials, and against various malignancies, and found to be capable of prolonging survival (Forde and Rudin, 2012; D'Arcangelo and Cappuzzo, 2013; Woo, 2015). The oral formulation of savolitinib, a highly selective type Ib MET inhibitor, has been approved by the China National Medical Products Administration for the treatment of patients with advanced non-small cell lung cancer (NSCLC) (Markham, 2021).

We found that savolitinib did appear to be associated with a series of adverse events (AEs) in relevant clinical trials in which its safety was investigated. Among the drug-associated AEs, nausea, fatigue, peripheral edema, and abnormal hepatic function were the most common (Lu, 2021; Wang, 2022). However, the incidence of severe allergic reactions (drug hypersensitivity, anaphylactic shock and hypersensitivity) was rare (Sequist, 2020). Herein, we report a rare case of shock, a serious adverse event, after treatment with savolitinib in an HIV-1–positive patient with advanced NSCLC.

## Case description

**FIGURE 1:**
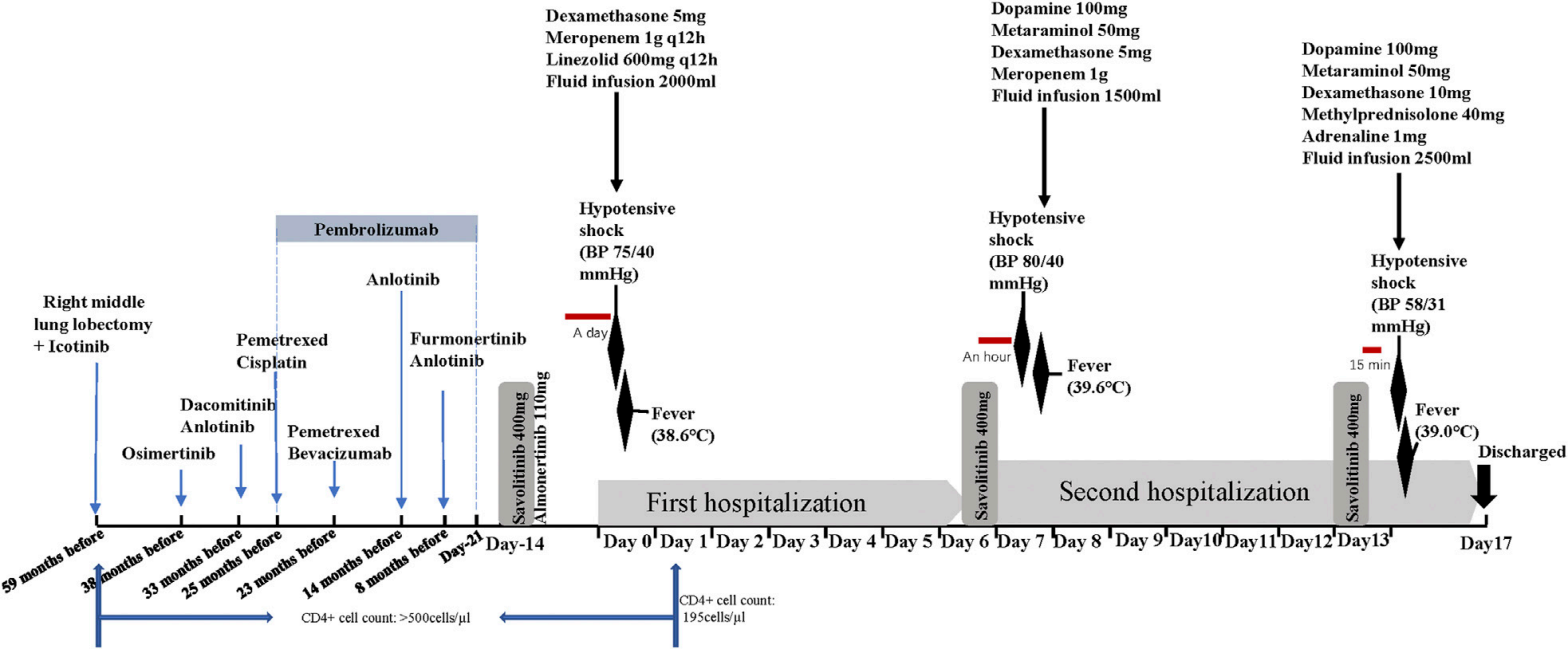
Treatment axis of the patient (time flows from left to right, day 0 is the time of the first adverse reaction).

The patient was a 38-year-old man with an 8-year history of HIV-1 positivity. His initial combination antiviral therapy (cART) regimen was lamivudine plus efavirenz plus tenofovir, and the CD4^+^ cell count remained >500/µL after the regimen. The cART regimen was eventually adjusted to bictegravir in January 2022, with good compliance. The patient was diagnosed with NSCLC 5 years ago and underwent right middle lung lobectomy and mediastinal lymph node resection under video-assisted thoracoscopic surgery in the year of diagnosis. Postoperative pathology confirmed poorly-to-moderately differentiated adenocarcinoma with lymph node metastasis, with a size of 3.0 cm × 2.0 cm, and tumor infiltration involved the lung membrane. Immunohistochemical staining was positive for PD-L1 (membrane +10%), TTF-1 (+), Ki-67 (+20%), Napsin A (+), and CK7 (+), and negative for ALK (−). Gene testing (next-generation sequencing, NGS) of tumor tissue sections showed an epidermal growth factor receptor (EGFR) exon 21 L858R mutation. The patient started taking icotinib. One and a half years after operation, pulmonary computed tomography (CT) reexamination revealed tumor recurrence in the right nodule and new lesions of the left lung, which were considered metastatic tumors. Although blood NGS revealed the T790M mutation was negative, the patient perferred to switch molecular targeted drug to osimertinib after completely discussion with his oncological doctor. Because the tumor unfortunately progressed repeatedly, he received various treatments, including immunotherapy, targeted therapy, and chemotherapy (Figure 1). Positron emission tomography–computed tomography (PET–CT) performed approximately 3 months ago (1 August 2022) suggested that the disease had progressed again. NGS of tumor tissues suggested EGFR L858R mutation (abundance: 54%) and MET amplification (copy number: 5.9), and the former was detected with 5.4% abundance in the blood sample. Based on the recommendations of oncologists, the patient began to take the third-generation EGFR-tyrosine kinase inhibitor (TKI) almonertinib (110 mg per day) and the MET inhibitor savolitinib (400 mg per day) from 1 August 2022. He habitually takes almonertinib in the morning and savolitinib at night.

Despite a mild rash after receiving savolitinib and almonertinib, the patient was not concerned. After 2 weeks of taking savolitinib and almonertinib, he was hospitalized with a fever. He denied a history of food and drug allergies. His CD4^+^ count dropped to <200 cells/μL. On admission (day 0), the patient had a temperature of 38.6°C accompanied by dizziness and chest tightness. He developed hypotensive shock, with blood pressure (BP) 75/40 mmHg and a heart rate >110 beats/min. The initial laboratory results were: C-reactive protein (CRP) level, 108.5 mg/L; white blood cell (WBC) count, 8.44×10^9^/L (93.4% neutrophils, 0.8% eosinophils); and lactate level, 2.8 mmol/L. Lung CT and B-ultrasound of the organs showed no signs of infection. Because of the patient’s septic shock-like symptoms, we added empirical antibiotics besides glucocorticoid for antishock therapy (concrete therapy shown in Figure 1). The hypotension resolved 7 h later. Savolitinib and almonertinib were discontinued. Four days later, the patient’s indicators of infection normalized. Further tests (such as blood culture, urine culture, and pathogen NGS) did not clearly reveal infection foci to confirm the diagnosis of septic shock. Hypotension did not develop during the subsequent hospitalization. On day 6, he was discharged from the hospital and asked to continue savolitinib and almonertinib.

However, he was readmitted because of a fever (39.6°C), nausea, dizziness, and palpitations approximately 1 h after oral administration of savolitinib on the night of hospital discharge. His BP was 80/40 mmHg, and laboratory examination showed a WBC count of 13.75×10^9^/L (97% neutrophils and 0.1% eosinophils) and lactate and CRP levels of 3.2 mmol/L and 108.6 mg/L, respectively. He received treatment similar to that administered on the previous hospital admission, and his clinical symptoms and inflammatory indicators resolved within a short time. We wondered if these were adverse reactions caused by savolitinib. Therefore, we asked the patient to take savolitinib on a trial basis after 1 week of continuous antibiotic therapy (i.e., on day 13). Just 15 min after taking savolitinib, he complained of chest tightness, shortness of breath, dizziness, and cold limbs. His BP was 58/31 mmHg, heart rate 142 beats/min, and temperature 39°C. He promptly received 10 mg dexamethasone, 40 mg methylprednisolone, and 1 mg adrenaline infusion. With continuous antishock treatment, the patient improved 1 h later. His BP gradually recovered to 127/55 mmHg, and his heart rate to 100 beats/min. Based on these three similar occurrences, we confirmed that these phenomena were savolitinib-related AEs. Although the lung CT indicated that tumor was shrinking and according to RECIST1.1, the patient was in partial response condition after taking savolitinib. In order to avoid the recurrence of life-threatening shock, crizotinib, instead of savolitinib, combined with almonertinib was the final targeted regimen according to oncologists’ recommendations. The patient did not experience similar adverse reactions over the next 2 months.

## Discussion

Herein, we present a case of a patient who developed resembling septic shock-like symptoms after taking savolitinib. Systemic examinations and the patient’s responses to treatment showed that savolitinib was the most likely culprit. To our knowledge, this is the first report of MET inhibitor-induced resembling septic shock-like presentations.

Lung cancer remains the leading cause of cancer-related deaths worldwide, with NSCLC accounting for 80% of cases (Servetto et al., 2022). Although the development of immunotherapy has greatly improved therapeutic effectiveness against NSCLC, its role in driving mutant NSCLC remains to be determined. Targeted therapy is currently considered to be one of the most promising options for managing the manifestations of mutations in carcinogenic drivers (López-Castro, 2022). Savolitinib, a type of MET-TKI, was approved for the first time in China in 2021 for the treatment of metastatic NSCLC (Markham, 2021). A multicenter phase II trial (NCT02897479) demonstrated that savolitinib monotherapy affords a good disease control rate and overall survival in patients with advanced NSCLC (Lu, 2021).

Regarding the known adverse effects of savolitinib, to date, severe systemic reactions resembling septic shock have never been reported. The main reason may be that most savolitinib-related studies included HIV-negative patients. Studies have established that HIV-positive individuals are more likely to develop drug reactions compared with the general population. Multiple heterogeneous factors are responsible for the substantial risk in HIV-infected patients, including polypharmacy, slow acetylator phenotype, glutathione deficiency, a CD4^+^ T Cell count of <200 cells/mm^3^ or >25 cells/mm^3^, latent cytomegalovirus and Epstein-Barr virus infections (Carr and Cooper, 2000). It was reported that the incidence of serious adverse drug reactions (sADRs) of trimethoprim-sulfamethoxazole in HIV-infected persons correlated with the HIV load (Coopman, 1993; Liu et al., 2018). Moreover, Rabaud et al. studied 592 HIV-infected patients who were first treated with cotrimoxazole during the Delta trial (a randomized, double-blind, controlled trial). They found that a low CD4^+^ cell count at the time of cotrimoxazole introduction was the only factor associated with the onset of sADRs (Rabaud, 2001). Our patient had an 8-year history of HIV-1 infection. Laboratory tests showed that his recent CD4^+^ count was significantly lower than that in previous measurements. HIV-infected patients with poor immune status may be highly sensitive to drug therapy (Greenberger, 2019). Therefore, we wondered whether the low CD4^+^ cell count was related to the circulatory shock induced by savolitinib.

Other possible causes of shock in this patient, such as sepsis, anaphylaxis, and severe immune-related AEs (irAEs), were also considered. The shock in this case had some similarities to septic shock (e.g., hypotension, fever, and elevated inflammatory markers). However, no causative infectious agent has been identified. Anaphylaxis is a rapidly evolving multisystem process involving the *epidermis*, lungs, and gastrointestinal and cardiovascular systems, which can lead to fatal airway obstruction, and ultimately cause hypoxemia and even shock (Simons, 2014; LoVerde, 2018). The classic anaphylaxis pathway is mediated *via* the production of IgE by T and B Cells and subsequent cross-linking of IgE with high-affinity receptors on mast cells and basophils (Nguyen, 2021). The serum IgE level of the patient did not increase. Other symptoms of anaphylaxis include airway edema, bronchospasm, eosinophilia, and vascular edema. However, these clinical events did not occur in this patient. Of course, other types of allergic reactions might occur through non-IgE–mediated pathways.

Cytokine release syndrome (CRS) is a rare and life-threatening immune checkpoint inhibitor (ICI)-related adverse reaction, which is caused by systemic immune dysregulation, immune cell overactivation, and cytokine release (Li, 2021). The clinical manifestations of CRS are fever, hemodynamic instability, and organ failure (Rotz, 2017). In most reported cases, CRS occurred within 2 weeks after the last ICI treatment (Ciner, 2021). Our patient exhibited fever and hypotensive shock 21 days after pembrolizumab discontinuation. The levels of cytokines such as interleukin (IL)-4 (4.42 pg/mL; normal <3.20 pg/mL), IL-6 (7.66 pg/mL; normal <2.90 pg/mL), and IL-10 (6.17 pg/mL; normal <5.00 pg/mL) were higher than the corresponding normal ranges in this case. These findings are common in patients with CRS. Nevertheless, the serum levels of these cytokines were very low compared with those previously reported for cases of CRS (Rotz, 2017; Dimitriou, 2019), and the serum IFN-γ and TNF-α levels were normal. The possibility of savolitinib triggering delayed and atypical CRS after the switch between immunotherapy and targeted therapy is interesting. The latency period for the three episodes progressively shortened and the symptoms continued to worsen. The pathogenesis of the circulatory shock was unclear; however, it was, to different extents, different from those of septic shock, anaphylactic shock, and CRS. Studies have established that HIV-positive individuals are a hundred times more susceptible to drug reactions than the general population, and advanced immunodeficiency portends an even greater risk (Hoosen, 2019). The symptoms clearly indicated the patient’s decreased immunity, whereby his CD4^+^ cell count dropped from >500 to <200 cells/μL. At present, it is unclear whether this phenomenon represents immune activation, drug toxicity, or a non-specific immune response, or appears independently in HIV-infected patients.

## Conclusion

To date, severe systemic reactions resembling septic shock caused by savolitinib have not been reported. To our knowledge, this is the first such case reported in an HIV-1–positive patient with advanced NSCLC. Savolitinib-induced symptoms that resemble septic shock are not a widely recognized phenomenon. However, they are worth considering in patients with immunodeficiency accompanied by circulatory shock who have recently taken savolitinib. It is critical to consider adding savolitinib to the allergy list of these patients.

## Data Availability

The original contributions presented in the study are included in the article/supplementary material, further inquiries can be directed to the corresponding author.
